# The cyanobacterial saxitoxin exacerbates neural cell death and brain malformations induced by Zika virus

**DOI:** 10.1371/journal.pntd.0008060

**Published:** 2020-03-12

**Authors:** Carolina da S. G. Pedrosa, Leticia R. Q. Souza, Tiago A. Gomes, Caroline V. F. de Lima, Pitia F. Ledur, Karina Karmirian, Jimena Barbeito-Andres, Marcelo do N. Costa, Luiza M. Higa, Átila D. Rossi, Maria Bellio, Amilcar Tanuri, Arnaldo Prata-Barbosa, Fernanda Tovar-Moll, Patricia P. Garcez, Flavio A. Lara, Renato J. R. Molica, Stevens K. Rehen

**Affiliations:** 1 D’Or Institute for Research and Education (IDOR), Rio de Janeiro, Rio de Janeiro, Brazil; 2 Institute of Biomedical Sciences, Federal University of Rio de Janeiro, Rio de Janeiro, Rio de Janeiro, Brazil; 3 Laboratory of Cellular Microbiology, Oswaldo Cruz Institute, FIOCRUZ, Rio de Janeiro, Rio de Janeiro, Brazil; 4 Department of Genetics, Institute of Biology, Federal University of Rio de Janeiro, Rio de Janeiro, Rio de Janeiro, Brazil; 5 Institute of Microbiology Paulo de Goes, Federal University of Rio de Janeiro, Rio de Janeiro, Rio de Janeiro, Brazil; 6 Academic Unit of Garanhuns, Federal Rural University of Pernambuco, Garanhuns, Pernambuco, Brazil; Fundaçao Oswaldo Cruz, BRAZIL

## Abstract

The northeast (NE) region of Brazil commonly goes through drought periods, which favor cyanobacterial blooms, capable of producing neurotoxins with implications for human and animal health. The most severe dry spell in the history of Brazil occurred between 2012 and 2016. Coincidently, the highest incidence of microcephaly associated with the Zika virus (ZIKV) outbreak took place in the NE region of Brazil during the same years. In this work, we tested the hypothesis that saxitoxin (STX), a neurotoxin produced in South America by the freshwater cyanobacteria *Raphidiopsis raciborskii*, could have contributed to the most severe Congenital Zika Syndrome (CZS) profile described worldwide. Quality surveillance showed higher cyanobacteria amounts and STX occurrence in human drinking water supplies of NE compared to other regions of Brazil. Experimentally, we described that STX doubled the quantity of ZIKV-induced neural cell death in progenitor areas of human brain organoids, while the chronic ingestion of water contaminated with STX before and during gestation caused brain abnormalities in offspring of ZIKV-infected immunocompetent C57BL/6J mice. Our data indicate that saxitoxin-producing cyanobacteria is overspread in water reservoirs of the NE and might have acted as a co-insult to ZIKV infection in Brazil. These results raise a public health concern regarding the consequences of arbovirus outbreaks happening in areas with droughts and/or frequent freshwater cyanobacterial blooms.

## Author summary

The uncontrolled spreading of cyanobacteria in drinking water reservoirs has been the cause of serious public health problems worldwide. Toxin-producing cyanobacterial blooms commonly occur during drought periods in the northeast (NE) region of Brazil. During Zika Virus (ZIKV) outbreak in 2015–16, Brazilian NE showed disproportionately higher microcephaly incidence. Here, we test the hypothesis that the cyanotoxin saxitoxin (STX) may act as a co-insult for ZIKV. Water quality surveillance data showed increased cyanobacteria population and higher STX amount in NE region during 2014–2018. *In vitro*, we observed that neural progenitor cell death was doubled after STX exposure to ZIKV-infected brain organoids. *In vivo*, chronic ingestion of STX during gestational period potentiated ZIKV-derived brain abnormalities in newborn mice. Our study provides new insights that may explain the discrepancies among Brazilian regions regarding CZS severity. Moreover, the data highlight the importance of cyanobacteria and cyanotoxin freshwater monitoring for future arbovirus outbreaks.

## Introduction

Human population growth, associated with disorderly occupation of territory, results in waste discarded in the freshwater reservoir. This environmental problem could be escalated by long periods of drought, leads to aquatic ecosystems eutrophication, with the main problem being the mass proliferation of cyanobacteria (blooms) [1]. Cyanobacterial blooms comprise hepatotoxin- and neurotoxin-producing species responsible for wild and domestic animals intoxication, besides the contamination of human drinking water supplies [2]. Previous studies have shown that 60% of all fresh water samples containing cyanobacteria used to be toxic, with neurotoxin-producing ones being more common in North America, Europe and Australia [3].

Brazilian northeast (NE) usually faces periods of severe drought, with the most severe ever recorded occurring between 2012 and 2016 [4]. Besides reducing the reservoirs to critical volumes, which results in water supply deficiency [5], this rainy scarcity favors cyanobacterial blooms [6,7]. A literature survey of publications about cyanobacterias between 1930 and 2016 showed that the highest number of toxic bloom events occurred in Pernambuco (PE) state, where was described the presence of microcystins, cylindrospermopsin, five variants of saxitoxin (STX) and anatoxin-a(S) in freshwater [8]. Health issues derived from cyanobacterial blooms have already been shown in Brazil, identified not only by minor symptoms (diarrhea, nausea, visual disturbance) but also death [2,9–11].

Extreme climate events promote changes in the dominance of cyanobacteria [7] as shown during the 1998 drought (a consequence of the El Niño in 1997), which favored the proliferation of *Raphidiopsis raciborskii* (formely *Cylindrospermopsis raciborskii*) [12] in almost 40 reservoirs in the NE of Brazil [6]. The *R*. *raciborskii* has high adaptability to unfavorable conditions because of its physiological characteristics, that includes akinete formation and tolerance to low phosphorus and nitrogen availability [13]. Most important, STX producing strains of *R*. *raciborskii* were positively selected among non-producing strains in NE superficial freshwater reservoir, as STXs would serve as a protection against water high salinity and or hardness [14–16].

The Brazilian strain of *R*. *raciborskii* produces STX, one of the most potent paralytic shellfish toxin (PST) found in freshwater and marine ecosystems [13]. PSTs are a group of neurotoxic alkaloids that act binding to voltage-gated sodium channels, blocking the generation of action potentials in neurons. The acute exposition to high amounts of PST results in numbness and even death by respiratory failure [17]. In contrast, little is known about the effects of chronic exposure to PSTs [18]. Because of their aforementioned dangerousness, a safety level of 3 μg/L of STX has been established in Brazilian water quality guidelines [19]. However, *in vitro* exposure to low levels of STX has already been reported to result in impaired neurite outgrowth and altered expression of proteins related to cell apoptosis and mitochondrial function [18,20].

The amount of STX usually found in reservoirs of the Brazilian semi-arid region varied between 0.003 and 0.766 μg/L, depending on the period of the year [21]. In 2000, during a toxic bloom at the northeast state of Rio Grande do Norte, *R*. *raciborskii* represented 90–100% of total phytoplankton species [22]. In case of severe water scarcity, the most impoverished population uses raw water from alternative sources without effective elimination of microorganisms. The consumption of water from ponds, water trucks, wells and household water reservoirs has already been associated with diarrhea outbreaks in states of the Brazilian NE [23]. Furthermore, it is important to notice that STX could also accumulate in marine organisms such as freshwater fish, which is the main animal protein source of many NE communities [24]. The effects of this accumulation in humans are not completely understood.

Zika virus (ZIKV) infection became an international concern when it was linked to a high rate of congenital brain abnormalities in Brazil [25,26]. The incidence of microcephaly varied among regions, with the highest frequency being found in the NE of Brazil [27,28] (S1 Fig). In contrast, the total number of cases of ZIKV infection was lower in NE compared to midwest or southeast (SE) regions [29]. Authors have suggested that a co-factor could be acting with ZIKV, contributing to this divergence among NE and other regions of Brazil; however, none has been confirmed until now [30].

The present study aimed to evaluate cyanobacteria and STX spreading among Brazilian regions during the ZIKV outbreak; We confirme a toxic synergism between STX and ZIKV *in vitro* using human brain organoids, and *in vivo*, using low-dose exposition of STX to mice that exacerbated the neurological consequences of congenital viral infection. Our results show that STX occurred in almost half of water analysis in the Brazilian NE, while the majority of other regions presented STX in less than 5%. STX combined with ZIKV increased neural cell death and brain malformations, *in vitro* and *in vivo*. Therefore, STX could be an environmental co-factor associated with the highest incidence of brain abnormalities caused by ZIKV in the northeast of Brazil compared to any other region of the world.

## Methods

### Occurrence of cyanobacteria and STX in water reservoirs of Brazil

The data about the number of cyanobacteria and STX presence were obtained from SisAgua—Water Quality Surveillance Information System for Human Consumption, a Brazilian Ministry of Health integrated data bank. The number of cyanobacteria per milliliter was determined in water reservoir destinated for human use, before treatment, from 2014 to 2018. Values were compiled and corrected by the number of municipalities in each Brazilian state. Then, the percentage of measurements below 10,000 cells/mL, between 10,000 and 20.000 cells/mL and above 20,000 cells/mL per municipality were organized per each region of Brazil. STX presence at treated water from 2014 to 2018 was compiled the same way as cyanobacteria concentration and their presence per municipality were organized per each region of Brazil.

### ZIKV propagation and titration

ZIKV (Recife/Brazil, ZIKV PE/243, number: KX197192.1) was provided by Dr. Marli Tenório Cordeiro from Fundação Oswaldo Cruz/Centro de Pesquisas Aggeu Magalhães, Brasil. The procedure of ZIKV isolation was described previously [31]. The virus was propagated in C6/36 *Aedes albopictus* cell line at a multiplicity of infection (MOI) of 0.01 and cultured for 6 days in Leibovitz’s L-15 medium (Thermo Fisher Scientific, Waltham, MA) supplemented with 0.3% tryptose phosphate broth (Sigma-Aldrich), 2 mM glutamine and 1x MEM non-essential amino acids (Thermo Fisher Scientific) and 2% fetal bovine serum (FBS). ZIKV titers were determined by conventional plaque assay.

### Human brain organoids

Human induced pluripotent stem (iPS) cells were obtained from Coriell Institute for Medical Research repository (GM79A). iPS cells were cultured in mTeSR1 media (StemCell Technologies, Vancouver, CAN) on top of Matrigel (BD Biosciences, Franklin Lakes, NJ). When colonies reached 70–80% confluence, iPS cells were dissociated with Accutase (MP Biomedicals, Santa Ana, CA), centrifuged at 300g, resuspended in media and counted. 9,000 cells/well were plated in ultra-low attachment 96-well plates and maintained at 37°C and 5% CO_2_.

Next day, medium was replaced with hESC media and embryoid bodies (EBs) were cultured for 6 days as previously described [32]. Then, EBs were transferred to 24-well ultra-low attachment culture plates containing Neural Induction Media: 1% N2 Supplement (Thermo Fisher Scientific), 1% GlutaMAX (Life Technologies), 1% penicillin/streptomycin (P/S), heparin 1 μg/mL for 4 days. Organoids were coated with Matrigel during 1 hour at 37 °C and 5% CO_2_ and returned to 24-well ultra-low attachment plates in Neurodifferentiation Media (NDM) with no vitamin A for additional 4 days in static culture and subsequently, transferred to agitation in NDM with vitamin A until day 50. Culture media changes were performed weekly.

### ZIKV infection in human brain organoids

The superficial cell number in organoids was calculated by dividing the superficial area (calculated using: 4πr^2^) by the mean cell area in the organoid surface (15 μm^2^). Brain organoids were infected using ZIKV MOI 0.5 (2–6.5 x 10^5^ PFU per organoid)—for 2 h, then cultured in medium with (or without) STX 12 μg/L (NRC Halifax, CAN) for 13 days. Mock-exposed organoids (treated and non-treated with STX) were used as control. The assay was performed in triplicates in three independent experiments.

### Animal experimental design, STX exposition and ZIKV infection

C57BL/6J ZIKV-refractory [33,34] nulliparous female 6-week-old mice received standard filtered water *ad libitum* supplemented (or not) with 15 ng/L of STX 7–10 days before mating and until harvesting date. All females were fed a standard diet with the recommended amount of macro and micro-nutrients (TD91352, Harlan Teklad, Madison). No significant differences in water intake were observed between groups.

Pregnancy was confirmed through observation of post-coital vaginal plug for estimation of embryonic age. ZIKV (virus plus C6/36 cell line supernatant) or Mock (C6/36 cell line supernatant) was administered intraperitoneally on E12. ZIKV groups received 10^6^ plaque-forming units per animal. Harvesting of samples was carried out on the first day of postnatal life (P0).

### Sample preparation for optical microscopy

Brain organoids and newborn mice brains were fixed in 4% paraformaldehyde solution (Sigma-Aldrich) for 2h and 48h, respectively. Organoids were cryopreserved in sucrose solution, immersed in O.C.T compound (Sakura Finetek, Netherland) and frozen at -80 °C, being sectioned at 20 μm slices in a Leica CM1860 cryostat for analysis. Newborn brains were embedded in 5% agarose/PBS (Bioline, Taunton, MA), being sectioned coronally at 80 μm in a vibrating microtome (VT1000S, Leica, Germany) for analysis.

### Immunofluorescence staining

After washing with PBS, sections were incubated in permeabilization/blocking solution (0.3% Triton X-100/ 3% goat serum, for organoids; 0.2% Triton X-100/ 2% goat serum, for newborn brains) for 2 h. For organoids, primary antibody rabbit IgG anti-Nestin (1:1000; RA22125 –Neuromics, Minneapolis, MN) was incubated at 4°C overnight. Apoptotic cells were stained with ApopTag Red in Situ (S7165, Merck Millipore) according to manufacturer’s instructions. For newborn brains, rabbit IgG anti-cleaved caspase 3 (1:300; 9661S - Cell Signaling, Danvers, MA) were incubated at 4°C overnight. Then, the sections were incubated with secondary antibody goat anti-rabbit AlexaFluor 488 (1:400; A-11008—Thermo Fischer Scientific) for organoids and newborn brains for 2 h. Nuclei were stained with 0.5 μg/mL 4′-6-diamino-2-phenylindole (DAPI) for 10 min.

For apoptotic cells quantification, 10–12 periventricular nestin-positive fields of 100 μm^2^ of three sections per organoid from each experimental group were analyzed. The number of TUNEL positive cells per area was quantified using Cell Profiler Software (BROAD Institute, Cambridge, MA). For nestin-positive area quantification, at least, 3 sections of individual brains organoids (3 per experimental group) were analyzed. The evaluation was performed in 3–5 independent experiments. Images were acquired on a confocal microscope Leica TCS SP8 and quantified with software Image J.

For the analysis of newborn brains, two sections per animal brain from each experimental group (three brains per group) were analyzed. Images were taken on microscope AxioImager A.1 (Zeiss, Oberkochen, DEU). All pictures were taken from the same correspondent cortical areas, in coronal sections at the level of anterior commissure crossing. Using the "straight" tool of Image J, we trace a line radially at the pallium-subpallium border from the pia-mater until the proliferative zone, through all layers of the cortex. Mock condition was used as control, and its line was copied and pasted in all images just as demonstration of cortex erosion. Caspase staining was evaluated quantifying the positive cells per cortical area described above.

### Ethical statements

Animals were housed in the Animal Care Facility of the Microbiology Institute of the Federal University of Rio de Janeiro. Protocols for animal handling were approved by the Research Ethics Committee of the Federal University of Rio de Janeiro (CONCEA registration number 01200.001568/2013-87, acceptance number 037/16).

### Statistical analysis

*In vitro* and *in vivo* results were expressed as mean plus standard error of the mean (SEM). Data sets were compared using One-Way ANOVA, followed by post-test of Dunnet with 95% confidence intervals, using GraphPad Prism Software. P-value < 0.05 was considered statistically significant.

## Results

### ~ 50% of analysis from water reservoirs in the Brazilian northeast had cyanobacteria and saxitoxin

**Fig 1:**
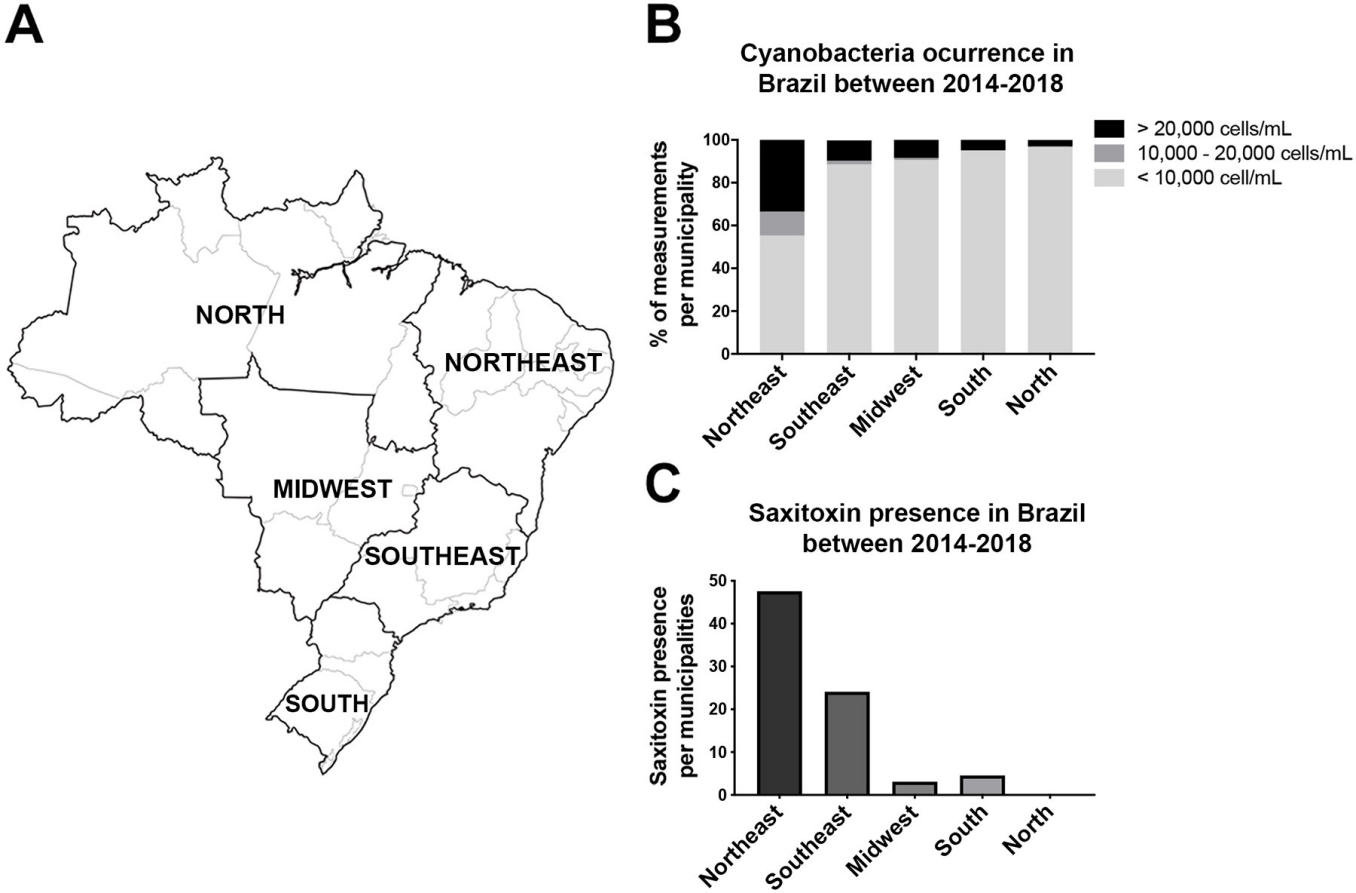
Cyanobacterial and STX occurrence among Brazilian Regions. (A) Brazilian map showing a division in regions. (B) Cyanobacteria in Brazil between 2014–2018. The measurements per municipality were split in ranges of cyanobacterial concentration. (C) STX 2014–2018 in Brazil. Note that NE had almost twice saxitoxin than SE.

Data describing the incidence of cyanobacteria in water reservoirs in Brazil were organized by the percentage of measurements per municipality in the concentration’s ranges: below 10,000 cells/mL, between 10,000 and 20,000 cells/mL and above 20,000 cells/mL. Measurements from municipalities were organized in regions according to the Brazilian Institute of Geography and Statistics (IBGE) classification, based on similar climate, geographic and socioeconomical aspects (Fig 1A). Between 2014 and 2018, NE showed ~ 34% of the measurements above 20,000 cells/mL, while other regions showed no more than 10% of the measurements on this range (Fig 1B–black bar).

The presence of STX per municipality was also evaluated. Half of NE water analysis showed STX in water reservoirs (Fig 1C–dark gray bar), followed by 25% in the SE (Fig 1C–medium gray bar). Other Brazilian regions presented STX in less than 5% of their municipalities (Fig 1C).

### Cell death induced by ZIKV was exacerbated by STX both *in vitro* and *in vivo*

**Fig 2:**
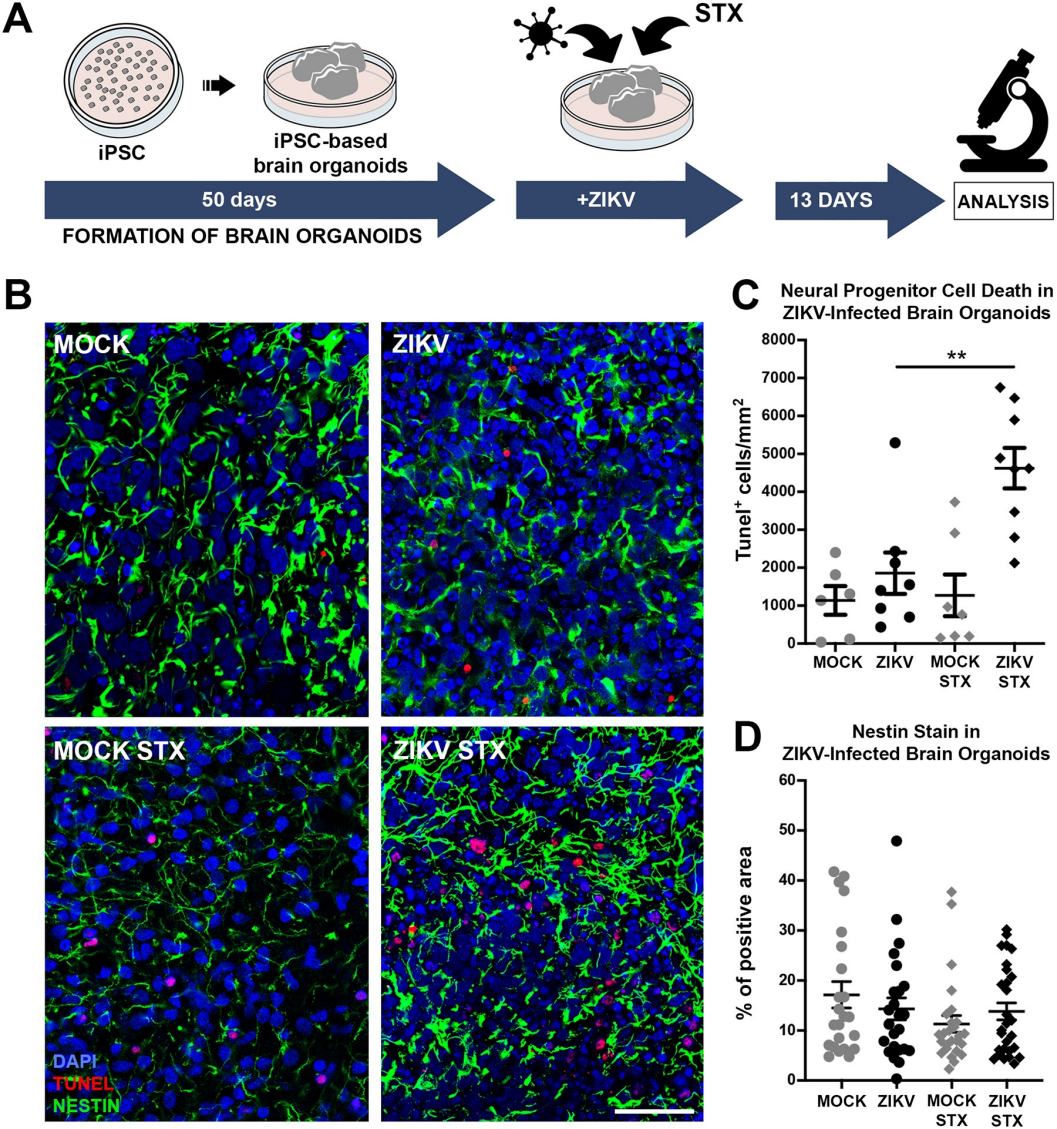
Saxitoxin increases cell death in ZIKV-infected brain organoids. 50-day-old brain organoids were infected with ZIKV and then exposed to STX for 13 days. (A) Summary of the experimental timeline. (B) Representative images of Nestin-positive areas (green) and TUNEL-positive cells (red) of untreated or STX-treated Mock and ZIKV-infected organoids. Scale bar means 50 μm. (C) Number of TUNEL-positive cells per Nestin-positive brain organoid areas (mean ± SEM). ANOVA, ** p < 0.01 (D) Percentage of Nestin-positive area per slices of brain organoid in the experimental groups (mean ± SEM).

In order to evaluate the effects of STX in the live human neural tissue, 50 day-old brain organoids were infected with ZIKV (MOI 0.5, which corresponds to 2 x 10^5^–6.5 x 10^5^ plaque-forming unit—PFU—per organoid) and then exposed to 12 μg/L of STX for 13 days (Fig 2A). This concentration of STX was chosen since it was often described in untreated water sources during droughts in the NE of Brazil [35]. Fixed organoids were sectioned in cryostat and immunostaining to identify apoptotic cells (TUNEL) and neural progenitors (Nestin) was performed. ZIKV-infected brain organoids exposed to STX presented ~ 2.5 times more dead cells per mm^2^ than ZIKV-infected organoids (Fig 2B and 2C). STX alone did not increase cell death in brain organoids (Fig 2C). The percentage of areas containing Nestin-positive cells was not significant different among the experimental groups (Fig 2D). To evaluate if STX changes ZIKV replication, supernatants of ZIKV-infected brain organoids were analyzed by quantitative PCR (RT-qPCR). STX increased ~ 3.4 times viral replication (S2 Fig).

**Fig 3:**
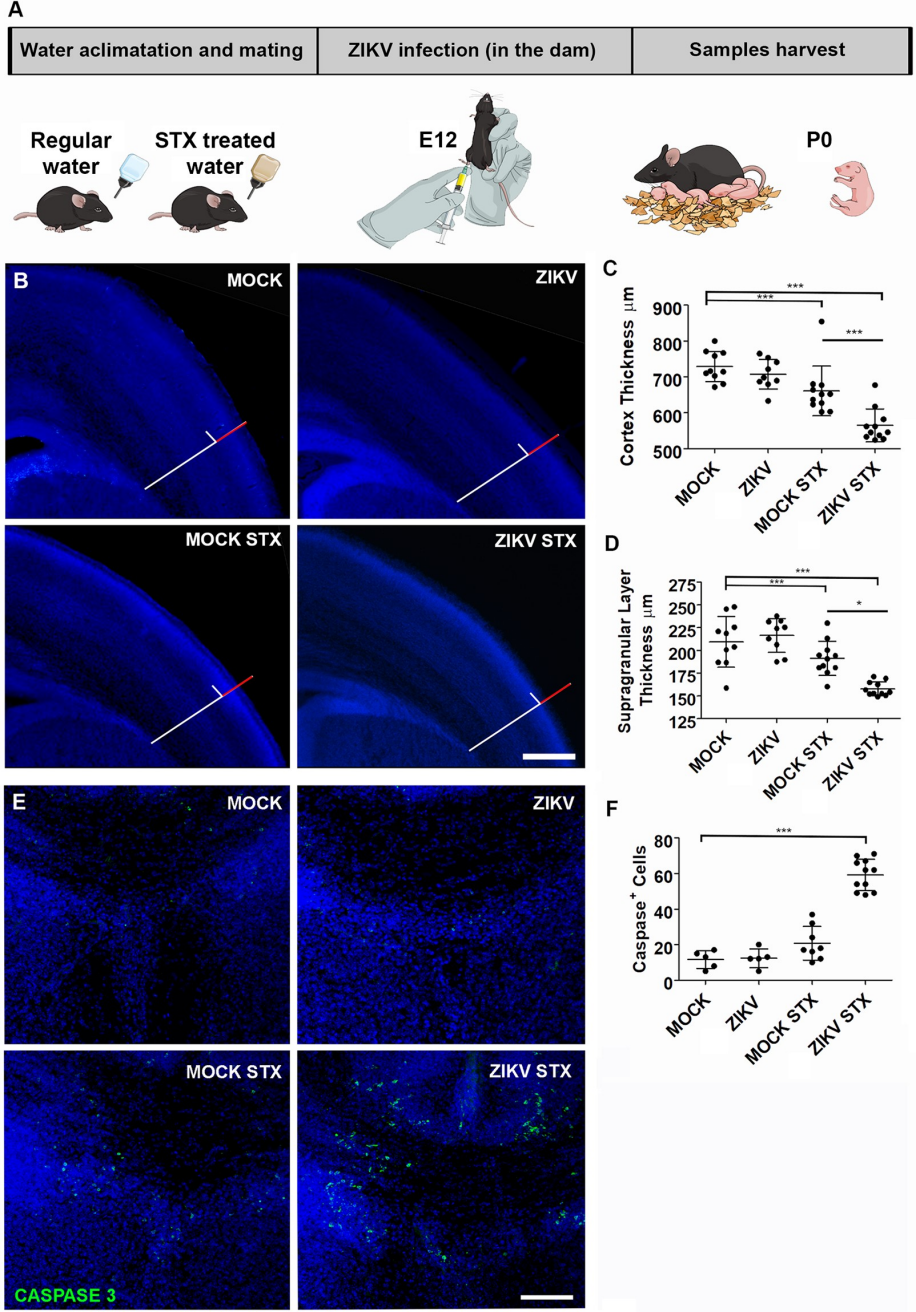
Mice offspring chronically exposed to STX during pregnancy presents congenital zika syndrome exacerbation. C57BL/6J pregnant mice continuously exposed to STX were infected with 10^6^ PFU of ZIKV intraperitoneally at E12. (A) Illustration showing the experimental timeline. (B) Representative images were taken from the same correspondent cortical areas, in coronal sections at the level of anterior commissure crossing. Nuclei were stained by DAPI. A reference line was traced radially in the Mock tissue, starting at the pallium-subpallium border from the pia-mater until the proliferative zone. Cortical erosion (red part of the line) was illustrated in the images based on the ZIKV STX image. Cortex (C) and proliferative supragranular cortical layers (D) thickness was measured among groups (mean ± SEM). (E) Representative coronal sections of the cortical midline where nuclei were stained by DAPI in blue and apoptotic cleaved-caspase 3 positive cells were stained in green. Scale bar means 400 μm (B) or 100 μm (E). (F) Quantification of caspase-positive cells per area (mm^2^) (mean ± SEM). ANOVA, *** p< 0.001, ** p< 0.01 and *p< 0.05.

To confirm *in vivo* the effects of STX as a cofactor of ZIKV neurotoxicity observed in human brain organoids, C57BL/6J mice were used. These animals, due to their efficient type I interferon signaling and ability to control ZIKV replication [33,34], do not present significant neurological impairments associated to vertical ZIKV transmission during embryogenesis [36]. Since the population of Brazilian NE is continuously exposed to STX (Fig 1C), and there is insufficient information about their cumulative effect, we decided to analyze the effect of chronic exposure to a low concentration of STX. We offered water contaminated with 15 ng/L of STX to immunocompetent C57BL/6J females one week before mating and continued during gestation. This concentration was chosen for being considered safe to humans and usually found in drinkable water in the NE, according to Brazilian government [19]. On gestational day 12, females were infected by intraperitoneal injection of 10^6^ PFU per animal. Offspring brains were analyzed on the day of birth (P0) (Fig 3A).

No difference in the distribution of Nestin-positive neural cells was observed among offspring (S4A Fig). ZIKV-infected females gave rise to mice presented mild cortical erosion, while ZIKV infected mice exposed to STX-contaminated water displayed a ~ 30% reduction in cortical thickness (Fig 3B and 3C). The size of infragranular layer (containing Ctip2 positive neurons) in animals exposed to both STX and ZIKV was reduced (S4B and S4C Fig). STX alone also reduced the thickness of the supragranular (Fig 3D) and infragranular (S4C Fig) cortical layers.

Additionally, to confirm if co-exposure of STX and ZIKV induce cell death in the developing cerebral cortex of mice, we quantified the number of caspase- positive cells in the cortical midline, where an accumulation of cell death was found. The amount of cell death in STX ZIKV-infected neonates increased more than twice, in comparison to other groups (Fig 3E and 3F). No increase in viral load was detected (S3 Fig). This apparent discrepancy may be explained by the fact that immunocompetent C57BL/6J mice is able to better control ZIKV replication [33].

Altogether, these data show that STX exacerbates cell death in both the progenitor zones of ZIKV-infected human brain organoids and in the brain of ZIKV-infected mice. Since the incidence of STX in water reservoirs was extremely high in the northeast, and it aggravates the neurogenic impairment caused by ZIKV both *in vitro* and *in vivo*, cyanobacteria may be considered a cofactor to the malformations caused by ZIKV in Brazil.

## Discussion

In the present study, we aimed to determine the participation of STX, one of the most neurotoxic and widespread PST naturally found, as a co-insult to ZIKV brain malformations. First, we showed that cyanobacteria and STX are notably prevalent in the Brazilian NE (Fig 1B and 1C), the region with the majority number of cases of ZIKV brain malformations described worldwide (S1 Fig). The evaluation of STX and ZIKV combined showed a two-fold increase in cell death (Fig 2B and 2C), while the chronic exposition to a lower concentration of STX in ZIKV-infected pregnant mice revealed a offspring with microcephaly-like phenotypes.

Issues related to drinking-water contaminated with cyanobacteria have already occurred in Brazil, United States and Australia [2,3,9,10]. Toxic cyanobacterial blooms commonly occur in the NE of Brazil, where large amounts of cyanobacteria and STX are common (Fig 1B and 1C). A recent study with cyanotoxin-contaminated water from the Brazilian NE showed a deep impairment of zebrafish development, including spine deformation and an increased rate of lethality [37]. A previous work showed that neuronal cells exposed to low doses of STX had inhibited axonal-like extensions, suggesting that cells remained in an immature state [18]. In human brains, ZIKV infects neural stem cells and glial cells rather than neurons [38].

Chronic exposure to STX before and during ZIKV-infection in mice, mimicked what might have occurred in the NE of Brazil. We offered water contaminated with 15 ng/L of STX to pregnant mice. This concentration is considered safe to human by Brazilian regulatory legislation [19] and is usually found in the drinkable water of the NE of Brazil, according to the SisAgua databank (Ministry of Health). Even in this concentration, significant impairment in cortical thickness (Fig 3B–3D) and increased cell death (Fig 3E and 3F) was observed in ZIKV-infected mice exposed to STX, similar to observed in zebrafish [37]. STX alone reduced the cortical thickness of offspring (Fig 3C).

While, STX increased ZIKV replication in the supernatant of brain organoids (S2 Fig), it did not alter ZIKV replication in fetal mice brain tissues (S3 Fig). This apparent discrepancy may be explained by the hability of the immunocompetent C57BL/6J mice to control ZIKV replication [33], which does not occur *in vitro*. Moreover, a dose-dependent effect of STX on ZIKV replication *in vivo* should also be considered. Oral administration could lead to less bioavailability of STX in the brain when compared to the observed *in vitro*.

STX itself blocks sodium voltage-gated channels, inhibits axonal-like extensions [18] and interferes with neural survival and synaptogenesis [39], we suggest that the synergism between STX and ZIKV increases cells death and severe malformations observed in the offspring mice exposed to both insults. These observations warn about the risks of the exposure to cyanobacteria during arbovirus outbreaks. It is important to clarify that microcephaly and other ZIKV-derived congenital abnormalities might be multifactorial. Therefore, other risk factors may have contributed to foster the uncommon pattern of CZS in Brazil [30]. ZIKV outbreaks occurred elsewhere; however, no epidemiological relationship between STX-producing cyanobacteria and congenital malformations derived from ZIKV infection was shown until now.

With this study, we shed light on the importance of governmental regulations for monitoring cyanobacterial blooms and their removal during water treatment, particularly on droughts. We also observed that STX may act synergistically with ZIKV even at concentrations considered to be safe by Brazilian authorities. Stringent standards and surveillance of drinking water in areas where ZIKV is reported will be critical for minimizing future harmful arbovirus-associated effects on human populations.

## Supporting information

S1 Fig Percentage of infants with microcephaly presence in brain images in NE and SE regions of Brazil. The comparative systematic review selected 37 manuscripts with brain images of infants with ZIKV-related malformations. The percentage of microcephaly-positive brain exams was placed in a representative map of Brazil, in which SE region is blue and NE region is red.
(TIF)

S2 Fig STX increases ZIKV replication in human brain organoids. (A) Representative images of ZIKV-positive cells (green) of untreated or STX-treated Mock and ZIKV-infected organoids. (B) Number of ZIKV copies per μL of supernatant of brain organoid (mean ± SEM). ANOVA, * p < 0.05.
(TIF)

S3 Fig STX does not increase ZIKV replication in fetal mice brains. (A) Representative images of ZIKV-positive cells (red) in mice brains. Unspecific NS1 antibody stain was observed on brain vessels. (B) ZIKV RNA quantified by RT-qPCR from P0 brain tissues (mean ± SEM). ANOVA, *** p< 0.001, ** p< 0.01 and *p< 0.05.
(TIF)

S4 Fig Mice offspring chronically exposed to STX and ZIKV during pregnancy showed similar distribution of Nestin^+^ neural cells and extensive reduction of Ctip2^+^ cell layer thickness. C57BL/6J pregnant mice continuously exposed to STX were infected with 10^6^ PFU of ZIKV intraperitoneally at E12 and pups were harvested at P0. Confocal microscopy images were taken from the same correspondent cortical areas, in coronal sections at the level of anterior commissure crossing. (A) Representative images for Nestin staining (red). Scale bar means 200 μm. (B) Representative images of Ctip 2^+^ (green) cell layer. Two parallels green lines were used to delimitate the boundaries of the cell layer. White lines show the cell layer thickness. Scale bar means 200 μm. (C) Quantification of Ctip 2^+^ cell layer thickness among the different experimental groups (mean ± SEM). ANOVA, **** p< 0.001, *** p< 0.005 and *p< 0.05.
(TIF)
