# Supplementary figures and images for: The cyanobacterial saxitoxin exacerbates neural cell death and brain malformations induced by Zika virus
Source: PLoS Negl Trop Dis. 2020 Mar 12;14(3):e0008060. doi: 10.1371/journal.pntd.0008060 (PMC7067372; doi:10.1371/journal.pntd.0008060)

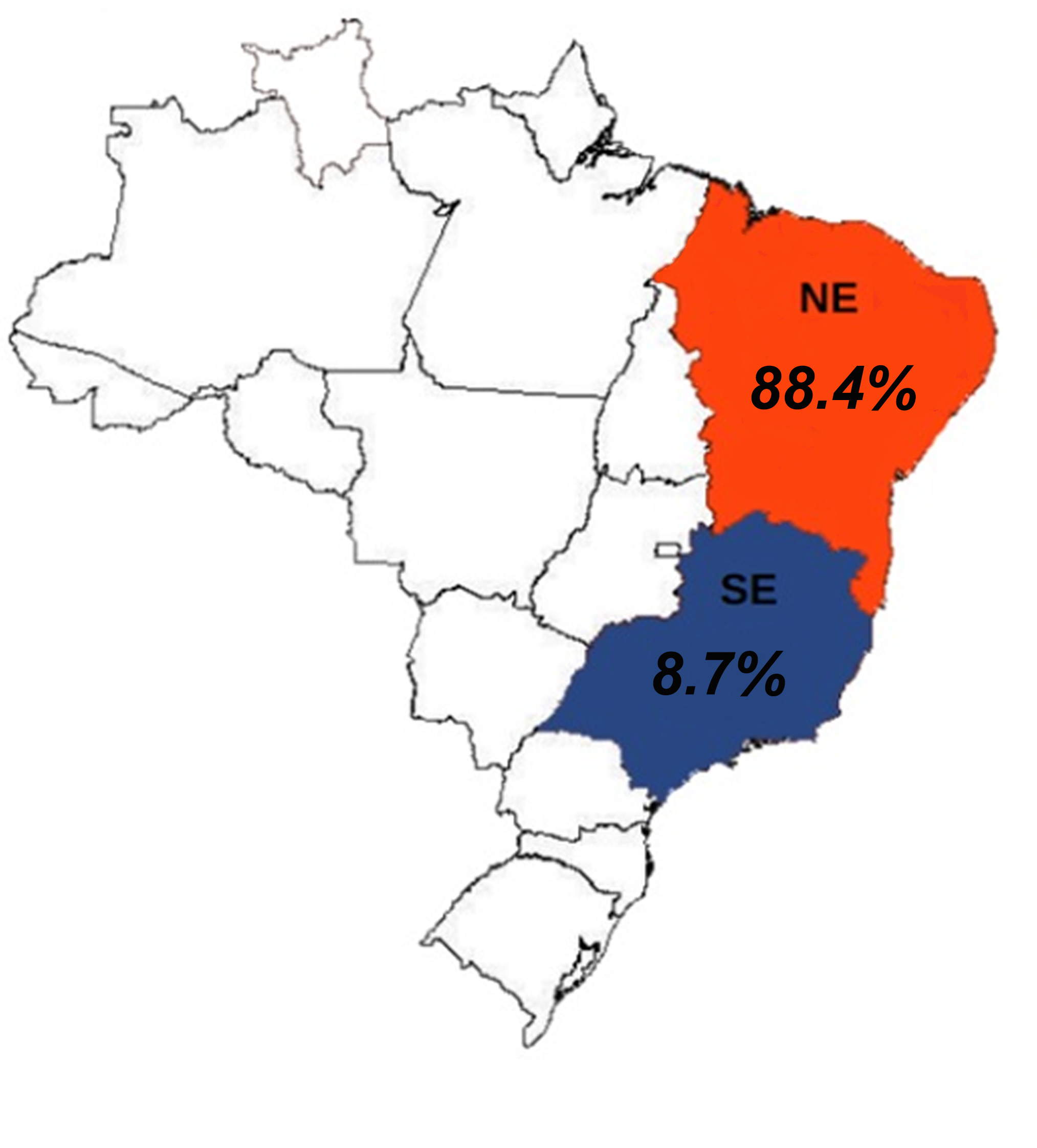

Supplement: S1 Fig — The comparative systematic review selected 37 manuscripts with brain images of infants with ZIKV-related malformations. The percentage of microcephaly-positive brain exams was placed in a representative map of Brazil, in which SE region is blue and NE region is red. (TIF) [file pntd.0008060.s001.tif]

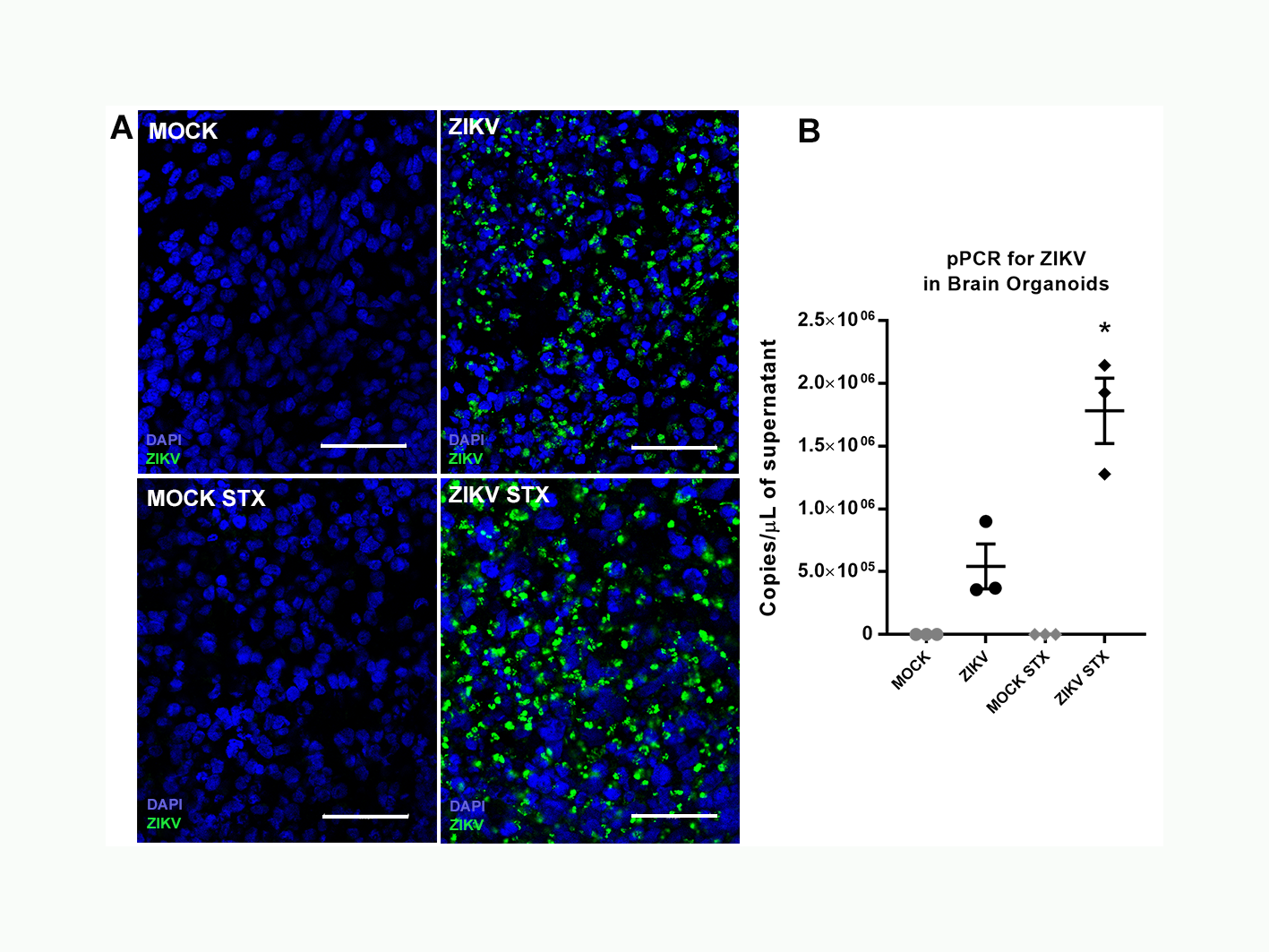

Supplement: S2 Fig — (A) Representative images of ZIKV-positive cells (green) of untreated or STX-treated Mock and ZIKV-infected organoids. (B) Number of ZIKV copies per μL of supernatant of brain organoid (mean ± SEM). ANOVA, * p < 0.05. (TIF) [file pntd.0008060.s002.tif]

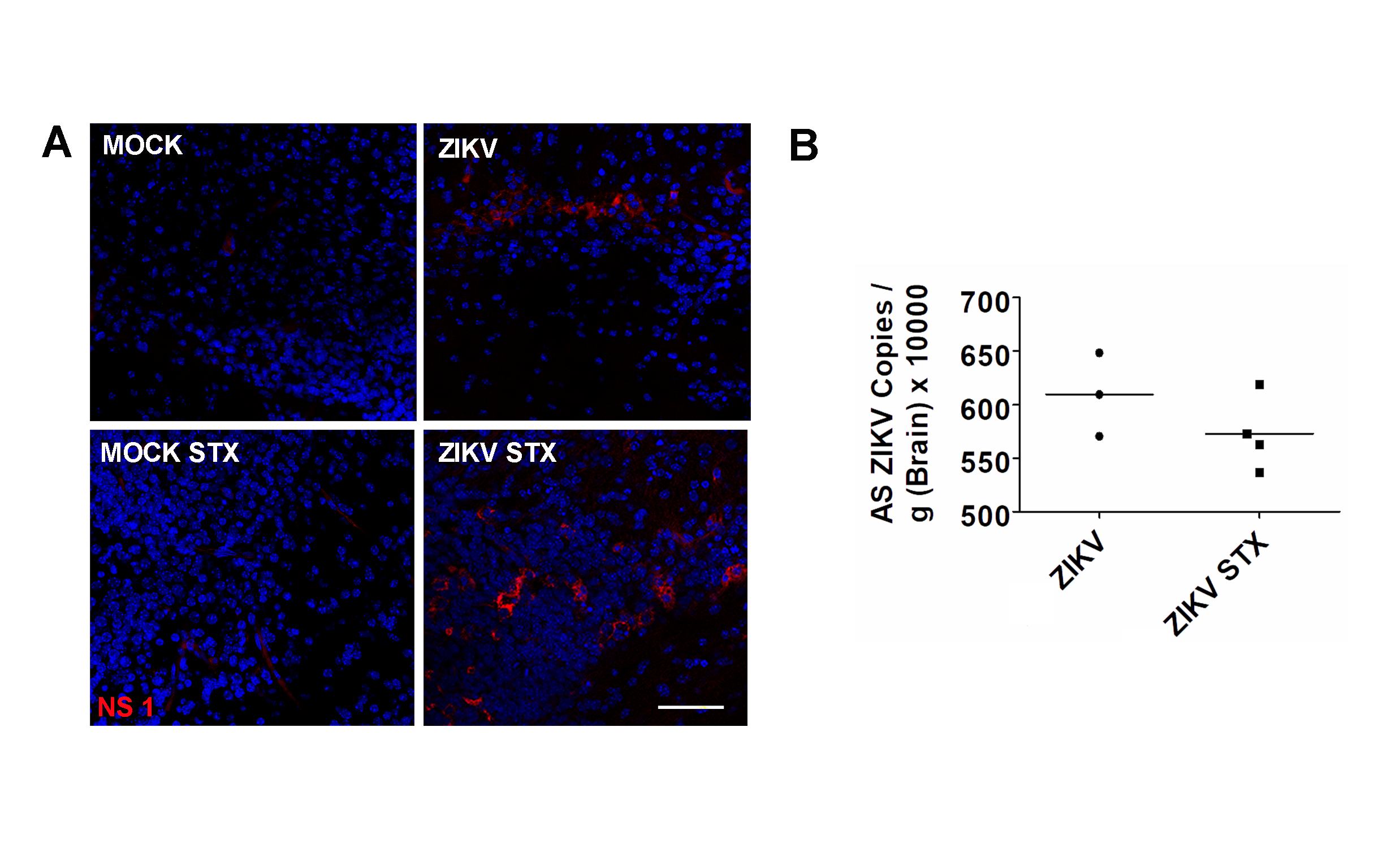

Supplement: S3 Fig — (A) Representative images of ZIKV-positive cells (red) in mice brains. Unspecific NS1 antibody stain was observed on brain vessels. (B) ZIKV RNA quantified by RT-qPCR from P0 brain tissues (mean ± SEM). ANOVA, *** p< 0.001, ** p< 0.01 and *p< 0.05. (TIF) [file pntd.0008060.s003.tif]

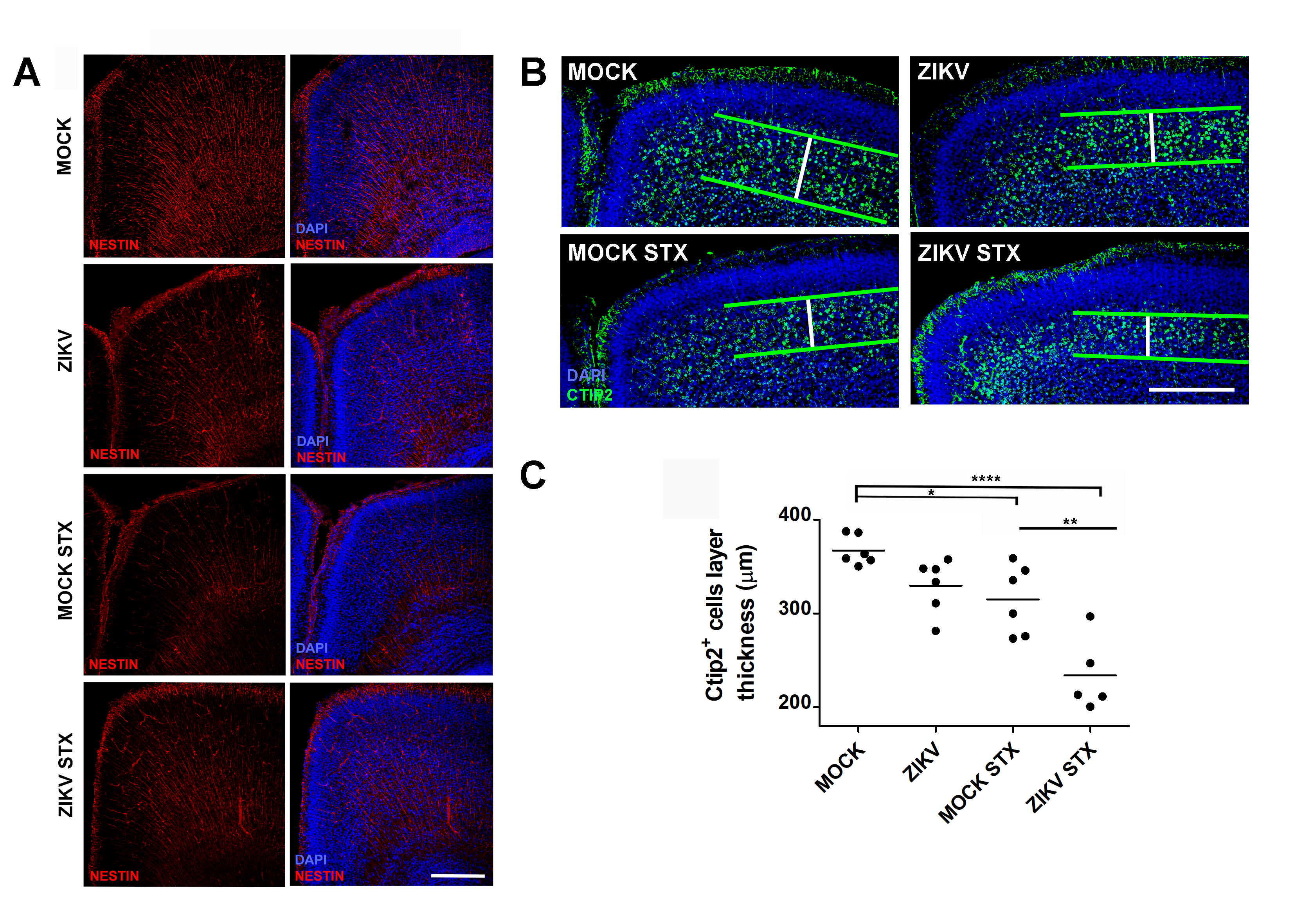

Supplement: S4 Fig — C57BL/6J pregnant mice continuously exposed to STX were infected with 106 PFU of ZIKV intraperitoneally at E12 and pups were harvested at P0. Confocal microscopy images were taken from the same correspondent cortical areas, in coronal sections at the level of anterior commissure crossing. (A) Representative images for Nestin staining (red). Scale bar means 200 μm. (B) Representative images of Ctip 2+ (green) cell layer. Two parallels green lines were used to delimitate the boundaries of the cell layer. White lines show the cell layer thickness. Scale bar means 200 μm. (C) Quantification of Ctip 2+ cell layer thickness among the different experimental groups (mean ± SEM). ANOVA, **** p< 0.001, *** p< 0.005 and *p< 0.05. (TIF) [file pntd.0008060.s004.tif]
